# Antimicrobial activities and mechanism of action of *Cymbopogon khasianus* (Munro ex Hackel) Bor essential oil

**DOI:** 10.1186/s12906-020-03112-1

**Published:** 2020-11-05

**Authors:** Gurpreet Singh, Meenu Katoch

**Affiliations:** Microbial Biotechnology Division, Indian Institute of Integrative Medicine, Canal Road, Jammu, 180001 India

**Keywords:** Antimicrobial, *Cymbopogon khasianus*, Essential oil, Minimum inhibitory concentration, Streptomycin

## Abstract

**Background:**

Due to concerns regarding the safety of the chemical control measures, the trend is shifting globally towards the use of natural compounds as antimicrobial agent especially, plant essential oils.

**Results:**

This study presented the antibacterial potential of *Cymbopogon khasianus* essential oil (CKEO) against human pathogens: *Pseudomonas aeruginosa*, *Salmonella typhimurium*, *Escherichia coli*, *Klebsiella pneumoniae, Bacillus subtilis*, *Staphylococcus aureus* and *Candida albicans* with MIC ranging from 20 to 100 μg/mL. CKEO, in comparison to its major constituent, geraniol, showed better MICs against tested pathogens. In combination studies, the effective concentrations of CKEO and streptomycin were reduced from 20 to 5 μg/mL and 11 to 0.7 ng/mL against *E. coli*. This suggests their synergistic action. However, CKEO showed partial synergy with ciprofloxacin. To understand the efficacy of CKEO, time-kill kinetics was performed*.* CKEO took the half time to show the bactericidal effect in comparison to streptomycin at their 2x MICs (double the MIC), while their combination took only 30 min for this. Fluorescence and surface electron microscopic and protein estimation studies suggested the multi-target action of CKEO-streptomycin combination against *E. coli*. Further, CKEO alone/in combination exhibited less than 10% haemolytic activity at its MIC.

**Conclusion:**

These results indicate that CKEO is a potentially safe alternative for the treatment of various pathogenic bacterial strains. It could be used for a variety of applications including human health, food storage, aquaculture, etc.

## Background

Antibiotic therapy is one of the most important therapies used for treating bacterial infections and has tremendously improved human life from the days of its introduction. But in the past few decades, the development and spread of resistant pathogenic strains threatens human health globally [1]. The most important factor for developing resistance is the excessive exposure of bacterial strains to antibiotics [2]. Consequently, it is essential either to reduce/halt the use of antibiotics or to develop and use the alternative of antimicrobials.

In recent years, interest in medicinal plants has attracted the attention of pharmaceutical and scientific communities as sources of antimicrobial substances. In the global market, due to concerns regarding the safety of the chemical control measures, the trend is shifting towards the use of effective and nontoxic antimicrobial agents amongst natural compounds that have been previously used in cosmetics, folk medicine, and aromatherapy [3, 4].

Essential oils (EOs), biosynthesized by aromatic medicinal plants as secondary metabolites, are the oily liquids that contain a mixture of compounds [5]. They possess various bioactive properties such as antimicrobial, antiviral, antimycotic, antioxygenic, antiparasitic, antiviral, and insecticidal [6, 7]. Initially, they were used because of their fragrance and flavour, but nowadays they are gaining popularity and widely used for a variety of purposes including household cleaning products, personal beauty care, prolonging the shelf life of food, in aquaculture and as natural medicines.

The Genus *Cymbopogon* (family Poaceae), is a tall densely tufted perennial grass with profuse tillering known for its medicinal use. Leaves of the plant were erect, green and linear. It is found either in wild or cultivated in tropical and semitropical areas of Asia, South and Central America, and Africa [8]. The leaves and roots of *C. olivieri* are widely used as antiseptic and for the treatment of stomach ailments [9]. Decoctions of the leaves and flowers of *C. giganteus* are effectively used for the treatment of migraine and diseases of skin, conjunctiva, and liver [10]. *Cymbopogon* essential oils have insect repellent properties for vectors of malaria, filariasis, and dengue [11]. Despite the immense commercial significance of the essential oils of the genus, little effort has been made on *C. khasianus* essential oil [12]. It is the right time to investigate the bioactive potential of *C. khasianus* essential oil when the demand for natural products is escalating because of its safe use with no side effects.

The aim of this research was to study the chemical profile and in vitro effect of the *C. khasianus* essential oil, alone and in combination with the commercial antibiotics, on the growth of pathogenic bacteria. Also, the possible mechanism of action was studied.

## Methods

### Plant material

*C. khasianus* was cultivated in the farmhouse of the Indian Institute of Integrative Medicine (IIIM, CSIR, Jammu, India) (Latitude 32^0^ 43′ N, Longitude 74^°^ 54′E, altitude 340 m asl). The plant flowers twice in a year (March to April and September to October). The identification of species was made by a taxonomist (Dr. S.N. Sharma, IIIM) and its specimen was deposited in the Janaki ammal herbarium, IIIM, Jammu with voucher number 3047. The leaves of plants (randomly selected twenty plants) were collected and used directly for the isolation of essential oil.

### Isolation of the essential oils

The fresh aerial parts of *C. khasianus* (1000 g) were subjected to hydro-distillation for 4 h in Clevenger type apparatus [13]. The distilled EO was dried over anhydrous sodium sulfate. The EO was stored in the dark at 4 °C until used for chemical analysis.

### Gas chromatography-mass spectrometry (GC/MS)

GC/MS analysis was carried out on a Varian GC/MS 4000 coupled with gas chromatograph 3800 fitted with a Varian factor four VF-5 ms fused silica capillary column. Its dimension were 30 m × 0.25 mm id, film thickness 0.25 μm. Temperature of the oven was set at 60 °C, which was programmed to rise to 250 °C at the rate of 5 °C/min. Helium was used as carrier gas. Its flow rate was set at 1 mL/min. Mass spectra were recorded with E.I. at 70 eV. Range of mass spectra was chosen 50–300 amu at the speed of one scan per second.

Initial volatile organic compounds (VOC) identification was made by comparison of obtained mass spectra of each component from CKEO with the mass spectra available in the NIST library (National Institute of Standards and Technology) database. Thus, the VOCs with ≥70% quality match score were presented in this report in the chemical terminology of the NIST database, while unidentified compound’s area were lumped together.

The final identification of the major compounds was done on the comparative chromatographic analysis of standard samples under similar conditions as described above. However, identification of several compounds was tentative due to non availability of their standards. Each compound present in CKEO was quantified approximately on the basis of its relative peak area obtained after GC/MS analysis. The GC/MS analyses were performed three times and mean values of relative peak areas were presented.

### Microorganisms

The antimicrobial assay was evaluated against human pathogens procured from Microbial Type Culture Collection, Chandigarh. The study includes both Gram-negative *E. coli* MTCC 118, *K. pneumoniae* MTCC 109, *P. aeruginosa* MTCC 424, *S. typhimurium* MTCC 98 and Gram-positive *B. subtilis* MTCC 121, *S. aureus* MTCC 737 bacteria and a yeast pathogen *C. albicans* MTCC 183. All the cultures were grown on nutrient agar media, while yeast extract peptone dextrose (YEPD) agar was used for *C. albicans*.

### Antimicrobial assay

The antimicrobial activity of the EOs was determined using the microdilution assay method [14, 15]. Briefly, each bacterial culture was aseptically inoculated in nutrient broth (HiMedia Biosciences) and grown overnight at 37 °C with shaking (200 rpm). The suspension was diluted to 1.5 × 10^8^ colony forming units (CFU/ mL) equivalent to 0.5 McFarland standard turbidity based on optical density (0.132) at the wavelength of 600 nm and finally adjusted to give approximately 1.5 × 10^5^ cells/mL for each organism. Fresh stock solution of CKEO (10 mg/mL) was prepared using DMSO. Different dilutions (10–100 μg/mL) were prepared from the stock solution of CKEO and different antibiotics (1 mg/mL in sterile distilled water). Antibiotics such as streptomycin, amphotericin B were used as positive controls. Different dilutions of CKEO and antibiotics (150 μL) were mixed with the nutrient broth containing 4 × 10^4^ CFU bacteria (50 μL). Appropriate negative (1% DMSO) and blank controls (un-inoculated media) were used. Microtiter plates (96 wells) were incubated at 37 °C for 12–16 h. The concentration, which killed 99.9% of the pathogen, was considered as the minimum bactericidal concentration (MBC), while the concentration, preventing the visible growth of bacteria under defined growth conditions, was considered as the minimum inhibitory concentration (MIC) [16]. MBCs were determined by plating 50 μL of the treated mix (without dilution) of the wells showing no colonies on nutrient agar.

### Assessing synergistic interaction between the essential oil, and different antibiotics (streptomycin, ciprofloxacin)

The combined effect of EO and conventional antibiotics (streptomycin, ciprofloxacin) on antimicrobial activity has been studied by the checkerboard microdilution test as described by [17] with few modifications. This assay was performed in a 96-well plate using two-fold serial dilutions. Conventional antibiotics were diluted vertically, whereas EO was diluted horizontally so that each well should contain different concentrations of two compounds. The starting concentration of each compound was the MIC. Following this, 50 μL suspension containing 4 × 10^4^ cells of test bacteria was pippetted to each well. The plates were incubated at 37 °C for 24 h and visually inspected for growth [16]. The lowest concentration of combination (EOs and antibiotics), which did not contain any visually detectable bacterial growth was considered as MIC. Besides, the control (containing test bacteria but no EOs/antibiotics) was used to assess the growth of bacteria, while the negative control was taken to assess the clarity/turbidity in the CKEO/combination. Each test was performed in replicates. The checkerboard generates many effective combinations (the first clear well of each row of the microtiter plate containing antimicrobial agents), and the fractional inhibitory concentration index (FICI) was calculated [18].

$$ \mathrm{FICI}={\mathrm{FIC}}_{\mathrm{C}\mathrm{KEO}}+{\mathrm{FIC}}_{\mathrm{antibiotic}}={\mathrm{C}}_{\mathrm{C}\mathrm{KEO}}/{\mathrm{MIC}}_{\mathrm{C}\mathrm{KEO}}+{\mathrm{C}}_{\mathrm{antibiotic}}/{\mathrm{MIC}}_{\mathrm{antibiotic}} $$

Where MIC_CKEO_ and MIC_antibiotic_ are MICs of CKEO and antibiotic when used alone; C_CKEO_ and C_antibiotic_ are MICs of CKEO and antibiotic at their isoeffective combination respectively. FICI was interpreted as follows: FICI < 0.5, synergistic, FICI of ≥0.5 and < 1 partial synergy; FICI of 1 additive; FICI > 4 antagonistic.

### Time kill kinetics

*E. coli* cells were grown to mid logarithmic phase. They were diluted as described above in antimicrobial assay. The diluted bacterial suspension (10^4^ CFU) was incubated with 1/2MIC, MIC and 2MIC concentration of oil/streptomycin or with 2FICI and 3FICI concentrations of their combination for different time period namely 10, 30, 60 and 120 min respectively. Post incubation, the cells were diluted by 10^2^ times and 50 μL suspension was spread on nutrient agar plates. Plates were incubated at 37 °C for 24 h and the colonies appeared on them were counted [15].

### Scanning electron microscopy (SEM)

Scanning electron microscopic studies were conducted to observe the changes in morphology of bacterial cells treated with antimicrobial agents [19]. *E. coli* cells were treated with streptomycin/EO/combination at their 2xMICs for 2–3 h. Then, treated cells were centrifuged and washed with sterile PBS solution. They were fixed with 2.5% glutaraldehyde in phosphate buffer (pH 7.4) for 24 h at 4 °C followed by washing and second fixation was done with 1% osmium tetraoxide for 6–7 h at 4 °C. Post fixations, dehydration of samples were done sequentially in ethanol of different concentrations (30, 50, 70, and 100%) for 30 min each. The drying of samples was achieved through hexamethyl disilazane/ethanol gradient series (50:50 and 100:0) for 30 min at each concentration. Finally, the samples were mounted on aluminium stubs and sputter-coated with gold, prior to inspection under microscope (JOEL JSM-6400, Japan).

### Fluorescence microscopy

To assess the effect of EO/antibiotic alone and in combination on bacterial membrane integrity, the double dye staining method was employed using DAPI (4′, 6 diamidino-2-phenylindole dihydrochlorides) and PI (propidium iodide) [15]. DAPI is a cell-permeable dye, which binds with A-T base pairs of double-stranded DNA and allows for powerful fluorescence signals, whereas PI is cell impermeable and only penetrates dead cells. The *E. coli* cells were grown up to mid-logarithmic phase and treated with streptomycin/EO alone and in their combination (at the 2x-MIC concentration) for 2 h at 37 °C. They were centrifuged at 5000 g for 15 min and the supernatant was removed. Pelleted cells were washed with sterile PBS solution then incubated with DAPI (10 μg/mL) and PI (5 μg/mL) respectively for 15–15 min at 0 °C in dark. After the dye treatment, cells were washed with phosphate buffer to remove excess stain. The cells without any antimicrobial agents were designated as control. Treated cells were observed under inverted microscope and their images were captured with the attached digital camera (Olympus Imaging Corp., Centre Valley, PA, USA).

### Protein estimation

Bacterial cells were grown up to mid-logarithmic phase as described above in antimicrobial assay and treated with MIC values of test compounds (CKEO/streptomycin alone and their combination). After the treatment of *E. coli,* the protein content in the extracellular medium was examined through colorimetric assay using a BCA protein assay kit (Thermo Scientific) as per the manufacturer’s instructions. The 96 well plate was kept in BOD set at 37 °C for 30 min, and absorbance was checked at 562 nm using a microtiter plate reader. The standard calibration curve was prepared using the bovine serum albumin (BSA) protein solutions (0–2 mg/mL) and used for calculating the protein content present in bacterial cell-extracellular medium with and without exposure of test compounds.

### The haemolytic activity

The haemolytic activity of CKEO, streptomycin alone and in combination was determined by using human red blood cells (RBCs) (blood used in the current study belongs to an author and was collected by the trained technician working in the dispensary of the Institute on his request) [20]. Fresh human RBCs were separated after centrifugation and rinsed thrice with sterile Phosphate buffer solution (PBS pH 7.4). The RBCs pellet was re-suspended in PBS solution to obtain a 5% solution. RBCs suspension (50 μL) was added to 96-well plate containing 150 μL of different concentrations of samples. The plate was incubated for 30 min at room temperature and was centrifuged at 2000 rpm for 20 min. The supernatant (100 μL) was transferred to a fresh 96-well plate and optical density (OD) was recorded at 360 nm wavelength. Triton X-100 (0.1%) was used as a positive control. The haemolysis was calculated in percent using a formula (sample abs –PBS abs)/ triton X abs- PBS abs)*100 [15]. The experiment was done in triplicate.

### Result and discussion

#### The essential oil analysis

The GC/MS analysis of the CKEO from the leaves of plants collected from the north-western region of India showed 26 components representing 98.25% of the EO with geraniol (81.74%), geranyl acetate (5.39%), cis-β-ocimene (3.02%), linalool (1.27%) and trans-β-ocimene (1.25%). (Table S1, Fig. S1). Choudhury and Leclercq [21] reported the same components in the CKEO from the leaves of plants with little variation in the percentage of geraniol (78.4%), geranyl acetate (7.3%), and linalool (2.2%) from the plants collected from the north-eastern region of India. The variation in the components of EO could be due to different eco-geographical regions. Some reports suggested that different varieties of *C. khasianus* collected/selected from north-eastern region of India contained different major compounds such as methyl eugenol/elemicin/geraniol in their EOs [22–24]. Recently, a study revealed that part of the plant (inflorescence/leaves) used for extraction of EO affects the yield of essential oil (0.82/0.70%), but not its components [12]. Similar to the current study, geraniol was found 18.81 and 22.78%, as the major component in the EO of *C. winterianus* collected from Brazil and Germany, respectively [25, 26]. *C. nardus* from Thailand contained geraniol (35.7%) as its major constituent, while *C. nardus* from Malaysia did not contain geraniol [27, 28]. Hence, in the recent past, a widest adaptable and stable variety (CIMAP Suwarna) of *C. khasianus* was chosen using AMMI (additive main effects and multiplicative interactions) model [29].

#### Determination of the antimicrobial activity of CKEO oil by microdilution method

The antimicrobial activities of CKEO and geraniol were examined against various pathogens using microdilution assay. Results in terms of both the minimum inhibitory concentrations (MIC) and minimum bactericidal concentrations (MBC) are given in Table 1. CKEO inhibited the growth of *E. coli* with MIC and MBC 20 μg/mL. It has also inhibited the growth of *B. subtilis, S. enterica typhimurium, S. aureus, K. pneumoniae* with MICs 25–50 μg/mL. The CKEO was also found active against *C. albicans* and inhibited the growth with MIC 100 μg/mL. These results suggested that CKEO had broad-spectrum antimicrobial activity against both Gram-positive and Gram-negative bacteria.

**Table 1:** Minimal inhibitory/bactericidal concentration (MIC/MBC in μg/mL) of *C. khasianus* essential oil (CKEO) essential oil and streptomycin

Pathogen	CKEO		Streptomycin/Amphotericin B		Geraniol
	MIC	MBC	MIC	MBC	MIC
*E. coli*	20	20	0.011	0.3125	> 100
*S. aureus*	30	–	–	0.3125	> 100
*P. aeruginosa*	20	30	–	0.3125	> 100
*S. enterica typhimurium*	30	–	–	0.3125	> 100
*B. subtilis*	25	–	–	0.3125	> 100
*K. pneumoniae*	20	–	–	0.3125	> 100
*C. albicans*	100	–	–	6.25	> 100

*E. coli* was found to be highly susceptible to CKEO with MBC of 20 μg/mL. The essential oil from *C. nardus* inhibited the growth of *E. coli* at much lower MIC (0.488 μg/mL) [28]. Bassole et al. [10] reported antimicrobial activity of EO of *C. citratus* and *C. giganteus.* Their MICs ranged between 2.1–80 mg/mL against *S. aureus*, *E. coli, P. aeruginosa,* and *S. typhimurium.* Adinarayana et al. [30] also observed growth inhibition of *S. aureus* and *B. subtilis* with the use *C. flexuosus* EO at a MIC of 250.5 μg/mL and 257.3 μg/mL respectively. Against *E. coli,* he reported MIC 294.5 μg/mL, which was higher than the present study. Sonboli et al. [31], reported the antimicrobial activity of the essential oil of *C. oliveri* with MIC of 3.75 mg/mL and 2.5 mg/mL against *B. subtilis* and *C. albicans,* respectively. Results suggested a high variation in antimicrobial activity of different species of the same genus might be due to the change in oil composition of the same/different species belonging to the different eco-geographical regions.

To understand the role of geraniol, a major constituent in CKEO, in antimicrobial activity, we compared the antibacterial activity of CKEO and geraniol against different pathogens. MICs of CKEO for all tested organisms were found to be lower than the MICs of geraniol. The MICs of geraniol in literature ranged between 0.5–1.4 mg/mL against different pathogens, which support the result of the present study [32–36]. Thus, it can be concluded that geraniol is not the sole responsible constituent for antimicrobial activity; other components such as geranyl acetate, linalool and β-ocimene are also involved in it [37–39]. In the contrary, methyl eugenol alone was found more effective against the microbes in comparison to methyl eugenol rich CKEO [40].

#### Synergistic interactions between the essential oil and antibiotics

Due to the non-availability of new antibiotics into the market and an increase in antibiotic-resistant pathogenic bacteria, alternative strategies are needed to cope up with the rising threat. In this study, the combined effect of CKEO with conventional antibiotics against *E. coli* was investigated by the checkerboard method, and the FIC and the FICI were calculated to determine the interaction of the CKEO with streptomycin/ciprofloxacin.

The CKEO showed total synergy in combination with streptomycin with FIC 0.316. However, its combination with ciprofloxacin showed partial synergy with FICI of 0.531.

Essential oils are a multi-component mixture with multiple target sites, due to which there is an increase in combination studies with conventional antimicrobial agents to treat infectious bacterial pathogens [41, 42]. Indeed, the combination of tested CKEO with streptomycin had shown significant results. The combination allowed a decrease in streptomycin concentration by 16 times, while there were four times decrease in the essential oil concentration (Table 2). However, in the combination, effective concentrations of ciprofloxacin and CKEO were reduced by two and thirty-two fold respectively.

**Table 2:** Minimal inhibitory concentration (MIC) of *C. khasianus* essential oil (CKEO) and streptomycin alone and in combinations against *E. coli*

Strain		MIC (alone)	MIC (μg/mL) combination	Fold reduction	Fractional inhibitory concentration index (FICI)	Result
***E. coli***	Streptomycin	0.011	0.0007	16	0 .316	Synergy
	CKEO	20	5	4		
	Ciprofloxacin	0.1875	0.0937	2	0.531	Partial Synergy
	CKEO	20	0.625	32		

**Fig. 1:**
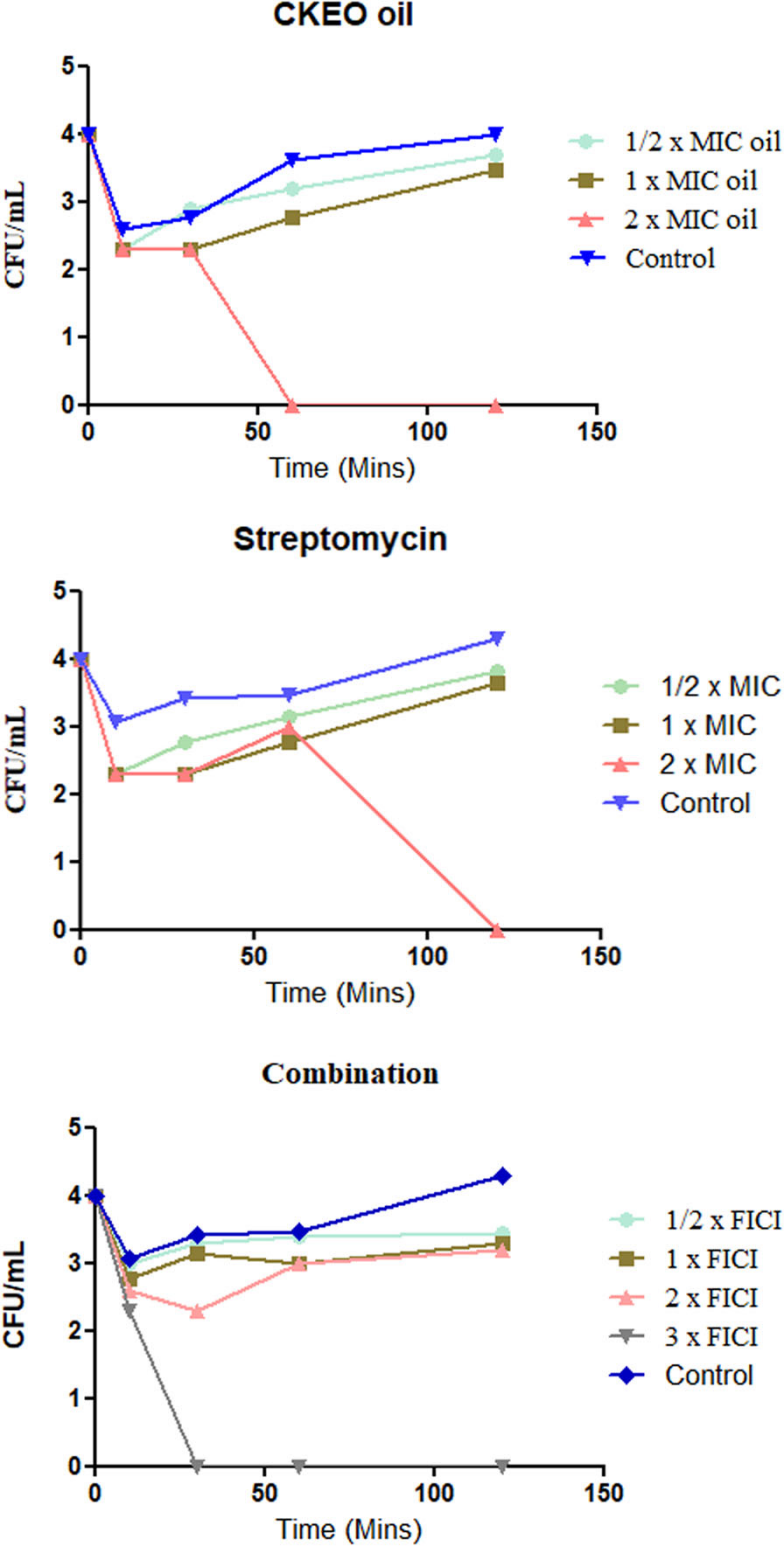
Time-kill curves of CKEO, streptomycin and their combination against *E. coli*. The count of dead cells was monitored for the first 2 h. Colour of lines indicates the concentration of the treatment used in the experiment blue untreated control, sea green 1/2 MIC/FICI, green 1xMIC/FICI, pink 2x MIC/FICI, grey 3xMIC/FICI

To understand the efficacy of CKEO and streptomycin alone and in combination, the time-kill assay was performed and the viable cell number in terms of CFU was calculated. The treatment with CKEO/streptomycin led to a dose-dependent response in terms of *E. coli* cell number (Fig. 1). At 2xMIC value, CKEO/streptomycin had totally eradicated the pathogen. At 2xFICI value, the combination of CKEO and streptomycin was found bacteriostatic but at 3xFICI value, pathogenic cells were completely eradicated within 30 min of exposure.

#### Scanning electron microscopy studies

**Fig. 2:**
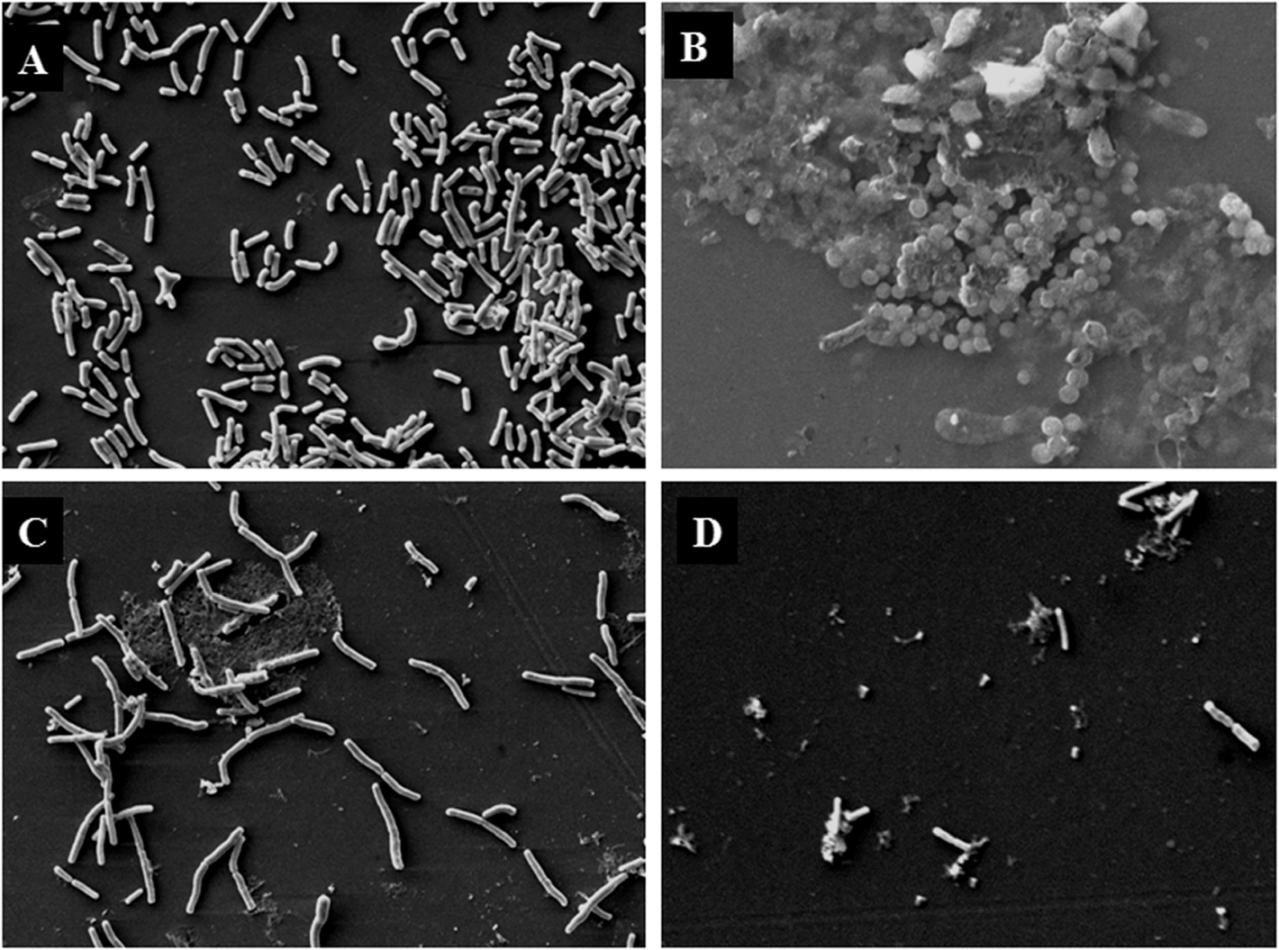
Scanning electron micrographs of *E. coli* (**a**) untreated cells (**b**) CKEO treated cells (**c**) streptomycin treated cells (**d**) CKEO plus streptomycin treated cells

The change in surface morphology of *E. coli* when treated at MIC concentrations of streptomycin (0.011 μg/mL), CKEO (20 μg/mL) and in the combination of streptomycin (7 ng/mL) plus CKEO (5 μg/mL) were examined. The control cells (when grown in the absence of any oil and streptomycin) under scanning electron microscope were found normal rod shaped structures with a smooth surface (Fig. 2). When treated with CKEO alone, small shrunken circular cells with wrinkled surface were observed, whereas with streptomycin treatment, cells became elongated. Cells treated with their combination had shown membrane shrinkage and destruction.

#### Fluorescence microscopy

**Fig. 3:**
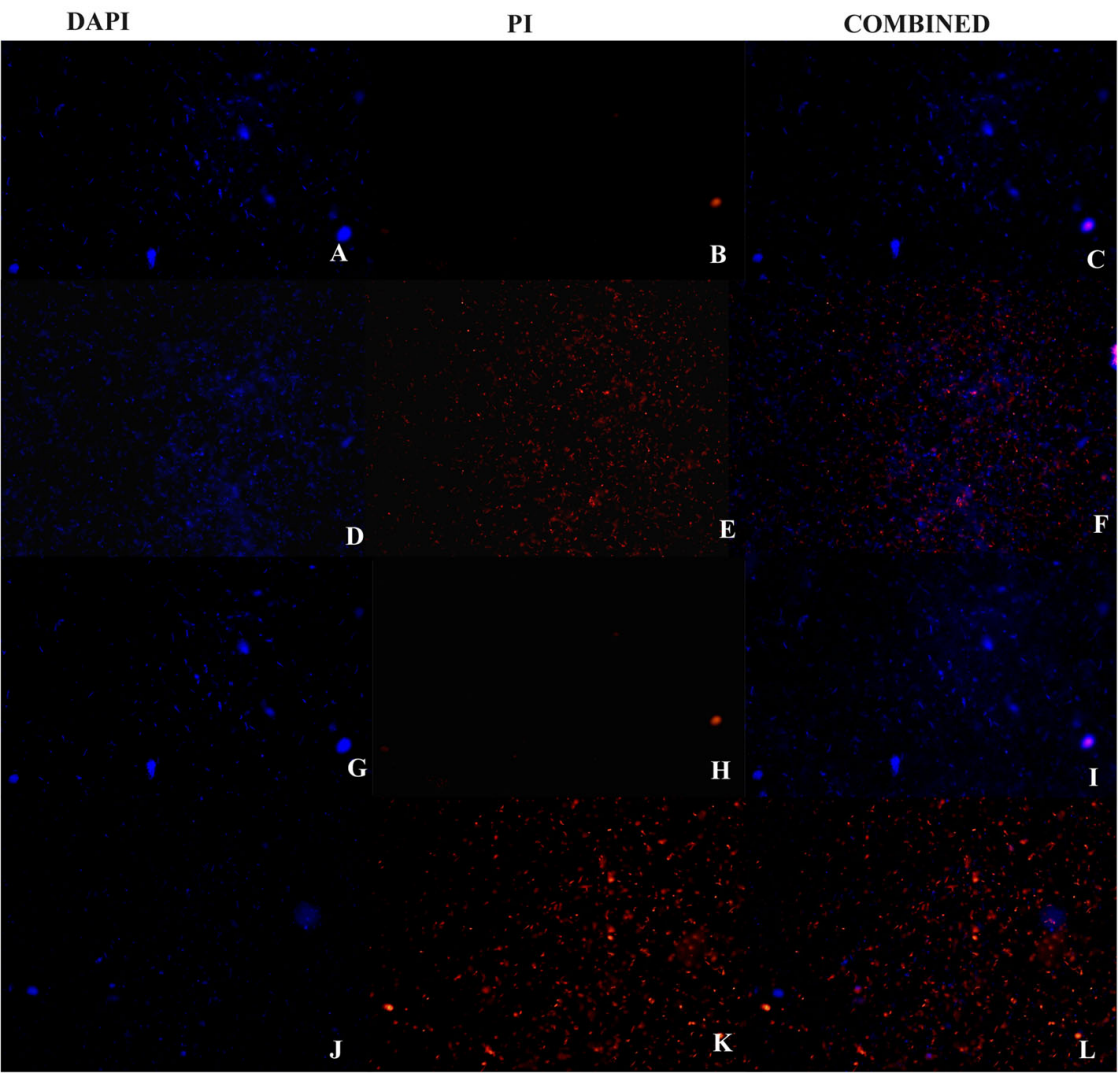
Fluorescence micrographs of *E. coli* cells. Untreated cells (A-C), CKEO treated cells (D-F), streptomycin treated cells (G-I) CKEO plus streptomycin treated cells

To study the effect of antibacterial agents on bacterial membrane integrity, fluorescence microscopy was employed [43]. As shown in Fig. 3, all bacterial cells in control were blue, whereas the treatment of cells with CKEO and their combination led both red and blue bacterial cells, suggesting it is a consequence of an impairment of membrane integrity. Hence the possible target of the CKEO is the bacterial membrane. Similarly, Zhang et al. [44] found that cinnamon oil altered the integrity and permeability of *E. coil* and *S. aureus* cells, while Heydari et al. [45] found that *Mentha piperita* and *M. arvensis* oils targeted the integrity of *Bacillus cereus* cell membrane. However, bacterial cells treated with streptomycin had shown only the blue signal (DAPI), which suggested that streptomycin does not compromise membrane integrity/permeability.

#### Protein content analysis

Protein content in the extracellular medium of untreated and treated *E. coli* cells was analyzed to understand the mechanism of CKEO, streptomycin, and their combination (Table 3). The analysis revealed the highest protein content in untreated *E. coli* cells. Dalbey and Kuhn [46] reported that many proteins, which got assembled into the inner and outer membranes of gram-negative bacteria, were exported to the periplasm or the extracellular medium. Cells treated with streptomycin showed 46% less protein. This might be because of many reasons: firstly the number of live cells in the treated sample would be significantly less than control. Secondly, the cell membrane of the dead cell is not damaged, which was already confirmed by florescent microscopy. Cells treated with CKEO showed more protein (20%) in comparison to streptomycin treated cells. As florescent microscopy study depicted that CKEO damages membrane integrity, which caused the release of proteins to present inside the cells. In the combination studies, (when *E. coli* cells were treated with one-fourth of CKEO and fifteen times less streptomycin) the protein content in the extracellular medium was found less in comparison to cells treated alone with CKEO, but more in comparison to cells treated alone with streptomycin. This protein content variation can be explained with results that streptomycin is not targeting cell membrane and inhibiting protein synthesis, which is a well-known fact. Hence, it might be involved in this interaction as well. Therefore, CKEO and streptomycin against *E. coli* acted in synergy but on multi-targets*.* Similar to our results, tea tree oil along with tobramycin, an aminoglycoside showed a synergistic effect against *E. coli* and *S. aureus* [47]. This was also an example of multi-target synergy.

**Table 3:** Protein Content analysis in extracellular medium of treated *E. coli* cells (mg/mL)

Treatment	Minimum Inhibitory Concentration (μg/mL)	Protein Content in extracellular medium of treated ***E. coli*** cells (mg/mL)	% protein inhibition in extracellular medium
Control	–	62.25	–
Streptomycin	0.011	33.58	46.05
CKEO	20	45.46	26.97
Streptomycin + CKEO	0.0007+ 5	43.46	30.18

*CKEO C. khasianus* essential oil

Further, CKEO, streptomycin and its combination exhibited less than 10% haemolytic activity at its MIC/FICI value, which suggested that it could be safe for humans (Table S2). Although, there is a trend of natural products, which are believed to be safer, still before use, in vivo studies and its toxicity related studies are to be conducted.

## Conclusion

These findings demonstrated that the essential oil obtained from *C. khasianus* is a potential alternative for the treatment of various pathogenic bacterial strains and its combination with streptomycin had synergistic interaction against *E. coli,* which can reduce the effective dose of streptomycin and CKEO both. Hence, in the future after properly addressing toxicity and safety-related issues, CKEO or its combination can be used in human health.

## Supplementary information

**Additional file 1: Table S1.** Chemical composition of essential oil of *C. khasianus* (Munro ex Hackel) Bor. **Figure S1.** GC-MS chromatogram of essential oil of *C. khasianus* (Munro ex Hackel) Bor. **Table S2.** Haemolytic activity of CKEO, Streptomycin and their combination at different concentration.

## Data Availability

Most of the data generated or analysed during this study are included in this published article and its supplementary file. The rest of raw datasets of bioactivity used and/or analyzed during the current study can be available from the corresponding author on reasonable request.

## Abbreviations

MIC: Minimum inhibitory concentration
MBC: Minimum bactericidal concentrations
CKEO: *Cymbopogon khasianus* essential oil
GC/MS: Gas Chromatography-Mass Spectrometry
VOC: Volatile organic compounds
NIST: National Institute of Standards and Technology
DMSO: Di-methyl sulfoxide
CFU: Colony forming unit
FICI: Fractional inhibitory concentration index
DAPI: 4′, 6 diamidino-2-phenylindole dihydrochlorides
PI: Propidium iodide
PBS: Phosphate buffer saline
RBCs: Red blood cells
