# Supplementary material for: Antimicrobial activities and mechanism of action of Cymbopogon khasianus (Munro ex Hackel) Bor essential oil
Source: BMC Complement Med Ther. 2020 Nov 5;20:331. doi: 10.1186/s12906-020-03112-1 (PMC7643435; doi:10.1186/s12906-020-03112-1)
Supplement: Supplementary file 1 — Additional file 1: Table S1. Chemical composition of essential oil of C. khasianus (Munro ex Hackel) Bor. Figure S1. GC-MS chromatogram of essential oil of C. khasianus (Munro ex Hackel) Bor. Table S2. Haemolytic activity of CKEO, Streptomycin and their combination at different concentration. [file 12906_2020_3112_MOESM1_ESM.docx]

**Antimicrobial activities and mechanism of action of *Cymbopogon khasianus* (Munro ex Hackel) Bor essential oil**

Meenu Katoch^1*^, Gurpreet Singh^1^, MK Verma^2^

^1^Microbial Biotechnology Division,

^2^Instrumentation Division,

Indian Institute of Integrative Medicine, Jammu, 180001, India

**Table S1:** **Chemical composition of essential oil of *C. khasianus*** (Munro ex Hackel) Bor

| **S. No.** | **Compounds** | **RT** | **% Identity** | **Relative peak area (%)** |
| --- | --- | --- | --- | --- |
| 1 | P-cymene | 6.026 | 77 | 0.14 |
| 2 | α-terpinene | 7.263 | 72 | 0.12 |
| 3 | L-phellandrene | 8.394 | 75 | 0.13 |
| 4 | Cis-β ocimene | 9.407 | 90 | 3.02 |
| 5 | Β-phellandrene | **10.823** | 80 | 0.37 |
| 6 | **Trans-β-ocimene** | **12.668** | **95** | **1.25** |
| 7 | 4-Nananone | 13.842 | 70 | 0.12 |
| 8 | **Linalool** | **24.230** | **97** | **1.27** |
| 9 | p-menth-2 en-1-ol | 32.315 | 93 | 0.75 |
| 10 | γ-terpinene | 33.048 | 90 | 0.5 |
| 11 | Unknown | 35.751 | <70 | 0.24 |
| 12 | Unknown | 37.216 | <70 | 0.26 |
| 13 | Nerol | 38.750 | 86 | 0.25 |
| 14 | Z-citral | 39.796 | 80 | 0.23 |
| 15 | **Geraniol** | **41.325** | **98** | **81.74** |
| 16 | Piperitone | 42.230 | 87 | 0.48 |
| 17 | E-citral | 46.213 | 75 | 0.36 |
| 18 | **Gerany acetate** | 51.983 | **91** | **5.39** |
| 19 | Β-elemene | 55.822 | 70 | 0.14 |
| 20 | Trans caryophyllene | 56.746 | 86 | 0.55 |
| 21 | Unknown | 59.460 | <70 | 0.17 |
| 22 | Germacrene D | 60.983 | 92 | 0.12 |
| 23 | Β-cadinene | 61.822 | 80 | 0.33 |
| 24 | Gerany butyrate | 63.746 | 90 | 0.9 |
| 25 | Geranyl ester | 65.428 | 91 | 0.36 |
| 26 | Others | - | <70 | 0.31 |

**Fig S1** GC-MS chromatogram of **essential oil of *C. khasianus*** (Munro ex Hackel) Bor

**
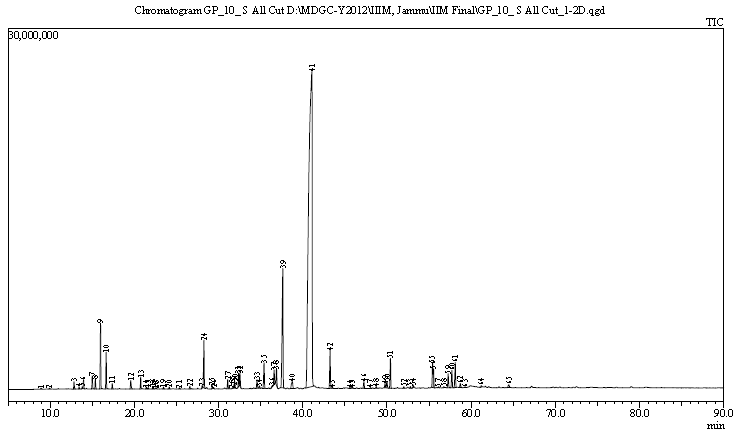
**

**Table S2: Haemolytic activity of CKEO, Streptomycin and their combination at different concentration**

| **Sample** | **MIC/FICI** | **Percent Haemolysis** |
| --- | --- | --- |
| CKEO | MIC | 7.2 |
|  | 2x MIC | 8.9 |
| Streptomycin | MIC | 4.2 |
|  | 2x MIC | 4.2 |
| Combination | 1x FICI | 5.8 |
|  | 2X FICI | 5.6 |
|  | 3X FICI | 6.8 |
